# Teledermatological Follow-Up Consultations in Individual Prevention of Occupational Dermatoses: A Monocentric Feasibility Study on Quality and Satisfaction by Patients and Physicians

**DOI:** 10.3390/ijerph20126127

**Published:** 2023-06-14

**Authors:** Carina Gill, Ann-Kristin Fischer, Katja Dicke, Björn Teigelake, Richard Brans, Christoph Skudlik, Swen Malte John, Cara Symanzik

**Affiliations:** 1 Department of Dermatology, Institute for Health Research and Education, Environmental Medicine and Health Theory, University of Osnabrück, Am Finkenhügel 7a, 49076 Osnabrück, Germany; ann-kristin.fischer@uni-osnabrueck.de (A.-K.F.); katja.dicke@uni-osnabrueck.de (K.D.); rbrans@uni-osnabrueck.de (R.B.); cskudlik@uni-osnabrueck.de (C.S.); sjohn@uni-osnabrueck.de (S.M.J.); cara.symanzik@uni-osnabrueck.de (C.S.); 2 Institute for Interdisciplinary Dermatological Prevention and Rehabilitation (iDerm) at the University of Osnabrück, Am Finkenhügel 7a, 49076 Osnabrück, Germany; 3 Berufsgenossenschaft für Gesundheitsdienst und Wohlfahrtspflege (BGW), Gesundheitscampus-Süd 29, 44801 Bochum, Germany; bjoern.teigelake@bgw-online.de; 4 Lower Saxony Institute of Occupational Dermatology at the University of Osnabrück, 49076 Osnabrück, Germany

**Keywords:** dermatosis, occupational, skin disease, teledermatology, telemedicine

## Abstract

Teledermatology has become very popular, and not only due to the SARS-CoV-2 pandemic. Patients with occupational skin diseases (OSDs) could also benefit from teledermatology services as part of their follow-up care, but the opportunities and challenges for patients and dermatologists, especially regarding quality and satisfaction, need exploration. In this single-center feasibility study, 215 patients taking part in a tertiary prevention program for OSD were invited to participate. After obtaining consent, a follow-up video consultation appointment with the center’s dermatologists was made. Quality and satisfaction with the consultations were evaluated by fully standardized online questionnaires filled in by the patients and dermatologists. A total of 68 teledermatological follow-up consultations were conducted by 10 dermatologists on 42 patients. Half of the dermatologists (50.0%) and 87.6% of the patients were satisfied with the video consultations. However, the lack of physical examination seems to be a problem, especially from the physicians’ point of view (75.8%). A total of 66.1% of the dermatologists and 87.5% of the patients saw video consultations as useful supplements to face-to-face consultations. The results of our feasibility study indicate general satisfaction of patients and physicians with teledermatological sessions in occupational dermatology, especially as a useful supplement to face-to-face consultation.

## 1. Introduction

Digitalization is advancing in all areas of medical applications, and e-health offers are constantly increasing [1,2]. The ban on remote treatment has been lifted, and makes it possible for physicians to provide advice and treatment exclusively via virtual communication media in individual cases, even to patients they do not yet know [3]. Such e-health offers are summarized under the term “telemedicine”, which is a sub-area of eHealth [1,4,5]. The term eHealth also includes the sub-areas of eAdministration, ePrevention, eResearch, and eLearning. It has already been shown that telemedicine services can improve physician–patient communication—especially in rural areas with poor medical infrastructure—and thus improve the physician–patient relationship [5,6,7,8,9,10]. With the outbreak of the SARS-CoV-2 pandemic at the beginning of the year 2020, the situation changed fundamentally and the demand for telemedicine services rose sharply, since thanks to telemedicine patients do not have to expose themselves to any risk of infection either on the way to or in the clinic, and can additionally save time and costs for transport to the clinic [4,10,11,12].

The area of telemedicine that deals with dermatoses is called ‘teledermatology’ [1,2,4]. In teledermatology, three different telemedical procedures are used: the store-and-forward system (SaF), the real-time procedure, and hybrid forms [1,4,8,9,11,13]. Previous studies and reports have shown that the diagnostic agreement rates between face-to-face and tele-consultations were almost the same [5,9,14]. Teledermatological consultations also played a key role in the treatment of several inflammatory skin diseases during the pandemic and can expand safe access to dermatological care in a cost-effective and efficient manner [11,15]. In addition, teledermatology has enabled physicians to treat their patients even if they live far away or are in quarantine [16].

Patients treated for OSDs in Germany primarily suffer from contact dermatitis of the hands, but a high number of patients with occupational skin cancer caused by natural ultraviolet (UV) radiation are also taken care of [9,17]. Occupational contact dermatitis of the hands can be categorized as irritant contact dermatitis (ICD) or allergic contact dermatitis (ACD) [18]. The treatment and overall management of patients with occupational contact dermatitis is provided under the dermatologist’s procedure (so-called Hautarzt-verfahren of the German Social Accident Insurance) in Germany [19]. As a part of this dermatologist’s procedure, the statutory accident insurance finances different prevention programs of a hierarchical multi-step intervention procedure, such as the outpatient secondary individual prevention (SIP) or the inpatient/outpatient tertiary prevention program (TIP) [20,21]. The TIP is offered to patients with severe or refractory skin diseases and consists of a three-week inpatient phase in a specialized center. Apart from intensified diagnostics and treatment, the patients undergo health education and psychological interventions to gain knowledge, increase motivation, and change attitudes and behavior towards the adequate use of skin protection and care in the workplace [20,21]. Moreover, adequate personal protective equipment is selected and afterwards provided to the patients. This is followed by a three-week outpatient phase with absence from work under the supervision of a local dermatologist. Afterwards, the patients ideally return to their workplaces with an improved skin condition and optimized skin protection measures. After the dismissal, patients are monitored through follow-up outpatient visits. Teledermatological consultations in the form of the video consultation have considerable potential for the reduction of face-to-face consultations in the monitoring of patients with OSDs [9]. However, in the field of teledermatology, there is still a need for research with regard to benefits and limitations, since no studies are yet available for a large part of dermatological diseases [2]. A few studies have investigated patient and physician satisfaction with and quality of teledermatological procedures, but very rarely in direct comparisons of the two groups and not specifically for patients with OSDs. Therefore, the aim of this feasibility study was to evaluate the quality and satisfaction of teledermatological consultations from the point of view of patients with OSDs and their dermatologists.

## 2. Materials and Methods

### 2.1. Study Design

This monocentric, evaluative feasibility study was conducted from June 2021 to June 2022 at the Institute for Interdisciplinary Dermatological Prevention and Rehabilitation (iDerm) at the University of Osnabrück, Osnabrück, Germany. Ethic approval was obtained by the Ethics Commission of the University of Osnabrück (procedure number 35/2021).

### 2.2. Participants

Adult female and male patients who took part in the TIP at the iDerm within the study period and who did not participate in another study involving face-to-face follow-up visits were invited to participate. Interested patients were provided with oral and written information by their dermatologist. Only patients who gave their written consent to participate in the study were enrolled. Exclusion criteria were age under 18 years, participation in another study, or missing written consent. All patients who denied participation within the framework of this project (teledermatological consultations) were offered standard face-to-face follow-up consultations as usual.

### 2.3. Teledermatological Consultation

In week three and week seven after the TIP, participants received a teledermatological consultation (i.e., online video consultation) with the dermatologist who took care of the respective patient during the TIP at our institute. This consultation substituted for the usual face-to-face follow-up consultation in our clinic (usually also three and seven weeks after discharge). Two days before the teledermatology appointment, a special link with a password for access to the open-source web conferencing tool BigBlueButton (BBB) (invokable GmbH, Remscheid, Germany) was sent via e-mail, with regulations for the device to be used.

The T3 follow-up examination usually marks the end of the 3-week working leave following the TIP and usually includes the question of whether the gloves recommended for the workplace during the TIP have already been supplied. Furthermore, the patient is asked about his or her skin condition (improved, stayed the same, worsened) and about the therapy during the preceding 3-week work absence. Finally, the patient’s skin condition is assessed by the dermatologist.

At the time of the T4 follow-up examination, the patient has usually already worked for 4 weeks, so one question during the examination relates to the fit/suitability of the recommended gloves for the workplace, whereupon follow-up counseling by a health educator may still be initiated. Furthermore, the patient is asked again about his skin condition under working conditions (improved, stayed the same, worsened) and about the current therapy. Finally, the patient’s skin condition is assessed by the dermatologist as in T3.

### 2.4. Questionnaire

Immediately after the teledermatological consultation, both patients and dermatologists received another email with a link to the fully standardized online questionnaire on LimeSurvey (LimeSurvey GmbH, Hamburg, Germany). Each patient was given a personal code that made it possible to compare the dermatologist’s and the patient’s evaluation for each session. The dermatologist who performed the video consultation was also informed of the corresponding code. Information on how to fill in the questionnaire was included at the beginning of the questionnaire. If patients had problems with the online questionnaire, they were offered the opportunity to fill out a written postal questionnaire and then received the questionnaire and a pre-paid return envelope by post. The pseudonymized questionnaire had four sections for the dermatologists and five sections for the patients, asking for: (i) sociodemographic characteristics (i.e., personal code, age, gender, education) and the tools used for the teledermatological session (i.e., device, speaker, microphone, help from others), (ii) evaluation of the importance of the teledermatological session (only in the patients’ questionnaire), (iii) rating of different statements about the quality of the consultation, e.g., “The use of video consultation technology is a useful addition to face-to-face counselling” on a 5-point Likert scale for agreement/satisfaction (1 = strongly agree, 2 = rather agree, 3 = neither agree nor disagree, 4 = rather disagree, 5 = strongly disagree), (iv) the technical devices used, and (v) a personal evaluation of the teledermatological session, including open questions, e.g., “What did you particularly like?”. The surveys for both groups are shown in Appendix A. The questions in section (iii) were based on the questions translated into German from the questionnaire on the satisfaction and usefulness of telemedicine, whose construct validity and internal consistency reliability have already been demonstrated for English and Spanish [22], and on questions from a survey on patient satisfaction with telemedicine in a prison [23]. Before the start of the actual feasibility study, a pretest was conducted with five TIP patients to check the comprehensibility of the questionnaire.

### 2.5. Data Analysis

Data analysis was performed with SPSS for Windows, version 27.0 (IBM Corp. Released 2020. Armonk, NY, USA: IBM Corp). The results of the descriptive data analysis are given as percentages (%) and mean values (M). Agreement ratings of ‘full agreement’ and ‘agreement’ were considered positive ratings.

## 3. Results

### 3.1. Participant Characteristics

Of the 215 patients who participated in the TIP between June 2021 and June 2022, 45 patients participated in another study involving face-to-face follow-up visits and were therefore excluded from our feasibility study. Another 123 patients gave no written consent. The reasons for non-participation are shown in Table 1 (more than one answer was possible).

A total of 47 patients gave written consent. No appointment could be made with five of these patients and they were thus subsequently excluded, resulting in 68 teledermatological sessions with 42 patients. Of the 42 participant patients, 17 patients (40.5%) took part in one teledermatological session, 24 patients (57.1%) in two teledermatological sessions, and one patient (2.4%) in three teledermatological sessions. Forty-eight questionnaires were completed by 32 patients after the teledermatology sessions (response rate of 70.5%), while 62 questionnaires were completed by the physicians (response rate of 91.2%). Only one patient (2.4%) requested and completed a written postal questionnaire. Of the respondent patients, 57.8% were female and the mean age was 45.0 years (standard deviation (SD) = 12.1). On the dermatologists’ side, 10 dermatologists participated in the feasibility study and performed a various number of teledermatological consultations (M = 6.2; SD = 4.6; range = 1–17). Of the dermatologists, 70.0% were female and the mean age was 40.9 years (SD = 8.5). All of the participating dermatologists were trained in occupational dermatology and familiar with the targeted patient population. The educational and occupational qualifications of the patient cohort as well as the devices used by the patients and physicians are shown in Table 2 and Table 3.

Among patients, the most commonly used device was a laptop (47.9%), while physicians mainly used a computer (96.8%). As a speaker, most patients (70.8%) used their integrated microphone of the used device. On the part of the dermatologists, 75.8% used a headset to communicate during the video consultation. For image transmission, most of the patients (83.3%) used the integrated device camera (45.8% integrated laptop camera; 25.0% integrated smartphone camera; 12.5% integrated tablet camera), whereas 96.8% of the dermatologists used an external webcam.

### 3.2. Quality and Satisfaction with Teledermatological Consultations from Patients and Dermatologists

During the teledermatological consultation, nearly half of the patients (44.2%) had help with the technical implementation and in 42.1% of the cases they were supported by a family member. Of the patients who had no support, 83.3% would not have wanted any help either.

Most (83.3%) patients felt good during their teledermatological session and the majority (81.2%) of the patients coped well with the online consultation platform BBB. Almost all (91.7%) of the patients rated the dialogue atmosphere in their teledermatological examination as good.

Almost half (46.6%) of the patients agreed that the consultation helped them to ease their problem, and of the physicians 72.6% agreed that the video consultation had helped their patients with their problem. The majority (87.5%) of the patients saw the teledermatological consultation as a useful supplement to the personal consultations, and in addition most (75.0%) of the patients found the video consultation just as satisfying as a face-to-face conversation; nevertheless, only 41.7% of the patients agreed with the statement that the teledermatological consultation could replace the personal consultation. Similar to this, only 37.1% of dermatologists agreed with the statement that teledermatological consultations can replace face-to-face consultations, and almost half (46.7%) of dermatologists consider talking to the patient during the video consultation to be as effective as a face-to-face consultation; more than half (66.1%) of the physicians see the video consultation as a useful addition to face-to-face counselling.

**Figure 1:**
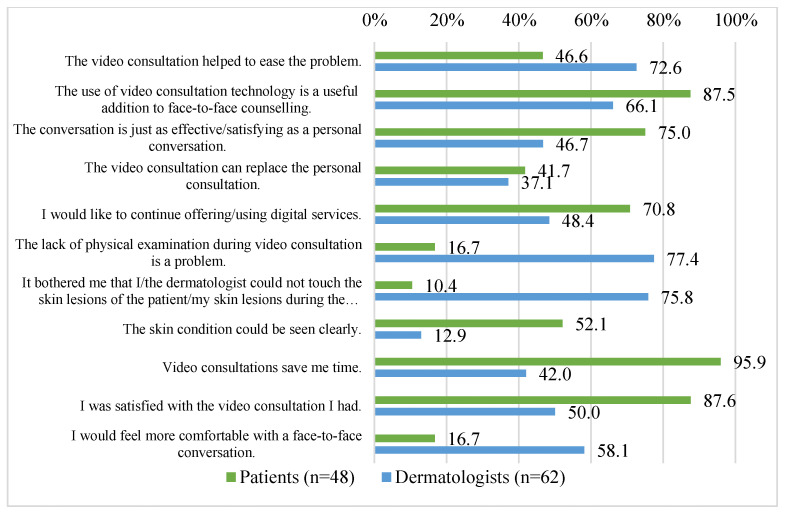
Quality of and satisfaction with teledermatological consultations. Ratings of various statements from patients’ questionnaires (n = 48) and dermatologists’ questionnaires (n = 62).

Most (70.8%) patients would use teledermatological consultations again, but only nearly half (48.4) of the dermatologists would like to continue offering digital services. Only 16.7% of the patients stated the lack of physical examination to be a problem and 10.4% of the patients were bothered that the dermatologist could not palpate their skin lesions, but most (77.4%) of the dermatologists agreed that the lack of physical examination was a problem and 75.8% were also bothered by the fact that they were not able to touch the skin lesions of their patients. Half (52.1%) of the patients stated that they believed their dermatologist was able to get a good picture of their skin condition, but only 12.9% of the physicians agreed to this statement. Asked about the time-saving factor of video consultations, almost all (95.9%) patients agreed that the teledermatological consultations were time-saving for them, while only 42.0% of the dermatologists agreed with this statement for themselves. The majority (87.6%) of the patients were satisfied with their teledermatological consultation; only 16.7% of the patients stated that they would feel more comfortable with a face-to-face conversation. Of the dermatologists, half (50.0%) of them were satisfied with the video consultation carried out, but 58.1% would feel more comfortable with a face-to-face consultation. These selected results are shown in Figure 1 and Table 4. The results of the questionnaires for all patients and dermatologists are shown in the Appendix B and Appendix C.

A striking result of the open questions was the insufficient image quality, which was complained about by the dermatologists in 27 questionnaires (43.5%). The answers to the open questions further show that 28.0% of the dermatologists had photos of the patient’s skin condition sent to them in advance or after the teledermatological video consultation in order to be able to more accurately assess it. The translated responses for the open-ended questions can be found in Appendix D.

## 4. Discussion

The aim of this feasibility study was to evaluate the quality and satisfaction of teledermatological consultations from the point of view of patients with OSDs and their dermatologists. The present study is, to the best of our knowledge, the first to focus on teledermatological consultations in this specific patient group. Our feasibility study has shown that most of the patients rated the quality of and satisfaction with video consultations positively, while dermatologists also rated it positively, but overall a bit lower.

The high satisfaction level of the patients was in line with other studies in the field of teledermatology [13,24,25,26,27,28], even though there are yet no comparable studies available in dermatological patients with occupational dermatoses. For example, Mostafa and Hegazy reported that 91.5% of patients considered their initial consultation via teledermatology to be equivalent to face-to-face consultations [12], while 75.0% of our patients considered their video consultations to be as satisfying as face-to-face consultations. These results are in contrast to the results of the study by Nicholson et al. in which 42.0% of patients with two-week-wait skin cancer referrals would have preferred face-to-face counselling [29]. However, our patients also saw barriers to teledermatology; only about half of the patients felt that their dermatologist was able to examine their skin disease well. This was also the finding of Pearlman et al., in whose study about half of the patients stated that their physician was only able to recognize their skin to an “excellent” or “good” degree, and several comments in the open-ended questions indicated that video consultation was better for follow-up consultations [13].

In our study, we were able to show that the dermatologists were satisfied with the video consultations, although only just less than half of them (48.4%) would want to continue offering video consultations. This result was in slight contrast to the results of Alakeel, who was able to show in his study that 67.8% of the dermatologists surveyed would continue to offer video consultations [30]. Kennedy et al. showed similar results in their study [31]. One reason for the discrepancy could be that 77.4% of the dermatologists in our study saw the lack of physical palpation as a problem and 75.8% were bothered by it. Other studies also regard the assessment of skin lesions solely on the basis of image documentation as critical [14,32,33]. Most of the patients in our study did not see a problem with this, which could be due to the fact that they probably did not know about the importance of the palpatory examination and felt very comfortable overall. Another reason could be that the patients in our study were already able to establish a relationship of trust with their physician during their three-week stay. Many of the dermatologists also complained about the poor image quality of the video consultations, which could be a second reason for the low interest in further video consultations. It was frequently stated in the open questions that the patient had been asked to send photographs of their skin condition in order to improve their assessment of the skin condition. A quick and easily feasible solution for the poor image transmission during the real-time video consultation could therefore be the combination of SaF and real-time video consultation. This would enable both the maintaining of personal contact and exchange with the patient and a better chance to assess the skin condition, which may improve the satisfaction of the dermatologist.

A large difference between patients’ and physicians’ answers could also be seen with regard to the time-saving factor of video consultations. While almost all patients agreed that video consultations saved them time, only 42.0% of dermatologists agreed with this statement for themselves. This could be due to the fact that the physicians were at their usual workplace during the video consultations, while the patients could save themselves the long journey. In addition, patients do not have to spend time in the waiting area, because they can do other things at home during the online waiting time. In future studies, it would be of interest to compare in more depth the time-saving factor between face-to-face and teledermatological follow-up consultations. Due to increased hygiene procedures (i.e., longer duration and frequency of hand washing), an increase in hand eczema has been reported in the general population and in high-risk occupations during the COVID-19 pandemic [34]. Such effects might have an additional impact on waiting times for patients, in which case teledermatology could be beneficial.

In addition, for our feasibility study it must be taken into account that this was a study with patients who were insured by the statutory accident insurance and who may have a different level of suffering or motivational background that prompts them to return to their former activity than patients with statutory health insurance. A comparison with patients with normal insurance, which could be conducted in future studies, would certainly be interesting.

There are some publications that address the opportunities and limitations of teledermatology in the field of OSDs from a theoretical perspective [9]. To our best knowledge, this is the first practical research in the field of OCD, especially ICD or ACD, that also compares patients’ and dermatologists’ perspectives on quality and satisfaction with teledermatological consultations. Another strength of our study is that the patients’ and dermatologists’ opinions could be directly compared for most items, whereas most studies only focus on the patients’ side.

Regarding limitations, a certain degree of selection bias is likely, as presumably it was mostly patients with sufficient technical possibilities or competences, or family support, and with enough motivation to try this technology, who participated in the teledermatological consultations and subsequently responded to the (online) study form. For older patients, a solely postal survey might have positively affected the response rate; however, it should be mentioned that this offer was made to all participants. Another limitation is the small sample size of our feasibility study and the described patient–physician bond, due to three week in-patient close monitoring prior to video consultation, which could bias the results. Furthermore, due to the relatively small sample size of our feasibility study, no statistical significance in the results was calculated, nor were any further statistical interferences found. It would be possible to investigate in larger studies whether and what influence the experience, age and gender of the dermatologists had on the respective assessment of the teledermatological examination of patients, which was not possible in our study due to the limited number of participating dermatologists. In general, further surveys are needed to establish the external validity of the presented results. As the quality of teledermatology is likely to be very dependent on many factors, namely the quality of the camera, microphone, and networks, it might be not sufficient to only classify the device by its kind (laptop, tablet, and smartphone). In future studies, further information on the cameras, microphones, and networks used could be assessed additionally to provide a more detailed overview.

## 5. Conclusions

To our knowledge, this is the first study to comprehensively evaluate patients’ and dermatologists’ satisfaction with and assessment of the quality of teledermatological consultations in the field of OSDs. In conclusion, the collected data indicated the general satisfaction of both patients and their physicians, especially in regard to interaction-related parameters, although the lack of physical examination seems to be a problem. Even though the overall quality of the video consultation technique was good, it could still be improved with better internet connectivity to enable concrete and satisfactory assessments of the skin conditions. Furthermore, in future teledermatological procedures, attention should be paid to minimum requirements regarding the quality of the technical equipment. Overall, video consultations seem to pose an appropriate consultation method in occupational dermatology, especially as a supplement to face-to-face consultations.

## Figures and Tables

**Table 1:** Reasons for non-participation in the study.

Reasons for Non-Participation	n	(%)
Participation in another study involving face-to-face follow-up visits	46	37.4%
Patient would prefer to attend the appointment in person	42	34.2%
Physician would prefer to follow up with patient in person	23	18.7%
Lack of technical equipment on the part of the patient	13	10.6%
Concerns about image quality on the part of the patient	10	8.1%
Patient lives nearby the clinic and wants therefore to come in person	10	8.1%
Poor internet connection on the part of the patient	8	6.5%
Concerns about the quality of the video consultation on the part of the patient	7	5.7%
Skin areas other than hands are affected	3	2.4%
No follow-up	2	1.6%
Language barrier on the part of the patient	2	1.6%
Patient has concerns about his technical competence	1	0.8%
Negative experience with online conferences on the part of the patient	1	0.8%
Not known	1	0.8%

**Table 2:** Educational and occupational qualification of the patient’s cohort.

Educational and Occupational Qualification n (%)	Patients n (%); Total of Patients: n = 32
Educational qualification n (%)	
No educational qualification	0
Secondary school/elementary school leaving certificate	7 (21.8%)
Intermediate school leaving certificate/secondary school leaving certificate	15 (46.9%)
Advanced technical college certificate	1 (3.1%)
General higher education entrance qualification/A-levels	4 (12.6 %)
Not specified	5 (15.6%)
Occupational qualification n (%)	
No occupational qualification	1 (3.1%)
Vocational training	18 (56.2%)
German ‘Master Craftsman’	6 (18.8%)
University degree	1 (3.1%)
Other occupational qualification	2 (6.2%)
Not specified	4 (12.6%)

**Table 3:** Devices of the study population.

	Video Consultations; Ratings by Patients (n = 48)	Video Consultations; Ratings by Dermatologists (n = 62)
Devices, n (%)		
Smartphone	12 (25.0%)	0
Laptop	23 (47.9%)	0
Tablet	6 (12.5%)	0
PC	4 (8.3%)	60 (96.8%)
Speaker, n (%)		
Headset	6 (12.5%)	47 (75.8%)
External Microphone	1 (2.1%)	12 (19.4%)
Integrated Microphone	34 (70.8%)	0
Webcam, n (%)		
External Webcam/Camera	3 (6.3%)	60 (96.8%)
Integrated Laptop Camera	22 (45.8%)	0
Integrated Smartphone Camera	12 (25.0%)	0
Integrated Tablet Camera	6 (12.5%)	0

**Table 4:** Selected results of the survey; comparison of physicians’ and patients’ questionnaires.

Ratings by Dermatologists (n = 62)					Ratings by Patients (n = 48)				
	Fully Agree		Rather Agree			Fully Agree		Rather Agree	
	n	%	n	%		n	%	n	%
The video consultation helped the patient to ease his/her problem.	21	33.9	24	38.7	The video consultation helped me to ease my problem.	8	16.7	14	29.2
The use of video consultation technology is a useful addition to face-to-face counselling.	23	37.1	18	29.0	The use of video consultation technology is a useful addition to face-to-face counselling.	28	58.3	14	29.2
The video consultation can replace the personal consultation with the patient.	14	22.6	9	14.5	The video consultation can replace the personal consultation with the dermatologist.	9	18.8	11	22.9
I would like to continue offering digital services, e.g., video consultations/online seminars.	15	24.2	15	24.2	I would like to continue using digital services, e.g., video consultations/online seminars.	22	45.8	12	25.0
The lack of physical examination during video consultation is a problem.	30	48.4	18	29.0	The lack of physical examination during video consultation is a problem.	3	6.3	5	10.4
It bothered me that I could not touch/feel the skin/skin lesions of the patient during the video consultation.	29	46.8	18	29.0	It bothered me that the dermatologist could not touch/feel my skin/skin lesions during the video consultation.	1	2.1	4	8.3
The conversation with the patient during the video consultation is just as effective as a personal conversation.	19	30.6	10	16.1	The conversation with the dermatologist during the video consultation is just as satisfying as talking to him/her in person.	18	37.5	18	37.5
Video consultations save me time.	22	35.5	4	6.5	Video consultations save me time.	32	66.7	14	29.2
I was satisfied with the video consultation I had with the patient.	7	11.3	24	38.7	I was satisfied with the video consultation I had with the dermatologist.	33	68.8	9	18.8
I would feel more comfortable with a face-to-face conversation.	15	24.2	21	33.9	I would feel much more comfortable with a face-to-face conversation.	3	6.3	5	10.4
I could clearly see the skin condition of the patient.	2	3.2	6	9.7	During the video consultation, the dermatologist was able to get a good picture of my skin condition.	15	31.3	10	20.8

## Data Availability

The datasets used and analyzed during the current study are shown in the Appendix A, Appendix B, Appendix C and Appendix D.

## Appendix A

**Table A1:** Questionnaires used in the present study.

Dermatologist Questionnaire	Patient Questionnaire
**Sociodemographic characteristics**	
What is your patient’s personal code?	What is your personal code?
How old are you?	How old are you?
Which gender do you feel you belong to? Male Female Diverse	Which gender do you feel you belong to? Male Female Diverse
	What school education do you have (highest qualification)? No educational qualification Secondary school/elementary school leaving certificate Intermediate school leaving certificate/secondary school leaving certificate Advanced technical college certificate General higher education entrance qualification/A-Levels
	What occupational qualification do you have (highest qualification)? No occupational qualification Vocational training German ‘Master Craftsman’ University degree Other occupational qualification
Which device did you use during the video consultation? Smartphone Laptop Tablet PC	Which device did you use during the video consultation? Smartphone Laptop Tablet PC
Which microphone did you use during the video consultation? Headset External microphone Integrated microphone	Which microphone did you use during the video consultation? Headset External microphone Integrated microphone
Which camera did you use during the video consultation? External webcam/camera Integrated laptop camera Integrated smartphone camera Integrated tablet camera	Which camera did you use during the video consultation? External webcam/camera Integrated laptop camera Integrated smartphone camera Integrated tablet camera
	Did you have support with the technical implementation of the video consultation? (e.g., logging in, activating access to camera/microphone, etc.)
	Who supported you in the technical implementation of the video consultation? Family member Dermatologist Other
	Would you have liked support for the technical implementation of the video consultation?
**Evaluation of the importance of the teledermatological session**	
	Which of the following services have you participated in? Three-week inpatient tertiary individual prevention at iDerm Osnabrück (TIP) Outpatient secondary individual prevention (ASIP) Skin consultation at a sch.uber.z Skin protection seminar at a sch.uber.z Treatment from a dermatologist Other
	How did you feel overall about the video consultation?
	How did you generally get on with the technology?
	How do you rate the atmosphere of the conversation?
	What is the significance of the video consultation for your treatment outcome?
**Quality of the consultation** **(5-point Likert scale for agreement/satisfaction)**	
The video consultation helped the patient to ease his/her problem.	The video consultation helped me to ease my problem.
I was able to advise the patient in a targeted manner.	I feel well advised.
I was able to provide comprehensive advice to the patient.	I felt well understood regarding my concerns.
I was able to fulfil my medical duty of care.	The use of video consultation technology is a useful addition to face-to-face counselling.
The use of video consultation technology is a useful addition to face-to-face counselling.	The video consultation can replace the personal consultation with the dermatologist.
The video consultation can replace the personal consultation with the patient.	I felt uncomfortable during the video consultation.
I was not comfortable doing the counselling via a video conferencing system (BigBlueButton).	I would like to continue using digital services, e.g., video consultations/online seminars.
I would like to continue offering digital services, e.g., video consultations/online seminars.	During the video consultation, the dermatologist was able to get a good picture of my skin condition.
The lack of physical examination during video consultation is a problem.	I was able to explain my (medical) problems well enough during a video consultation.
It bothered me that I could not touch/feel the skin/skin lesions of the patient during the video consultation.	The lack of physical examination during video consultation is a problem.
The privacy of the patient is protected during the video consultation.	It bothered me that the dermatologist could not touch/feel my skin/skin lesions during the video consultation.
The conversation with the patient during the video consultation is just as effective as a personal conversation.	My privacy was protected during the video consultation.
Video consultations make it easier for me to contact the patient.	The conversation with the dermatologist during the video consultation is just as satisfying as talking to him/her in person.
Video consultations are a convenient form of health care for me.	Video consultations make it easier for me to contact the dermatologist.
Video consultations save me time.	Video consultations are a convenient form of health care for me.
I was satisfied with the video consultation I had with the patient.	Video consultations save me time.
I would feel more comfortable with a face-to-face conversation.	I was satisfied with the video consultation I had with the dermatologist.
	I would feel much more comfortable with a face-to-face conversation.
**Quality of technology/technical equipment** **(5-point Likert scale for agreement/satisfaction)**	
The video consultation technique worked.	The video consultation technique worked.
The video consultation technology is easy to use.	The video consultation technology is easy to use.
My technical equipment made it difficult to understand the patient.	My technical equipment made it difficult to understand the dermatologist.
Due to the technical equipment of the patient, I had difficulties hearing him/her.	The devices made me feel uncomfortable.
The devices made me feel uncomfortable.	I could see the dermatologist well.
I could see the patient well.	I could hear the dermatologist well.
I could clearly see the skin condition of the patient.	I have an internet connection which, in principle, allows me to participate in video consultations.
The internet connection was stable during the video consultation.	The internet connection was stable during the video consultation.
I could hear the patient well.	The information sheet helped me to get to terms with the video consultation technique.
In general, I am satisfied with the video consultation system.	
**Personal evaluation of the teledermatological session**	
What do you think could be done better?	What will you tell others about the counselling?
What did you particularly like?	What do you think could be done better?
What else would you like to tell us?	What did you particularly like?
	What else would you like to tell us?

## Appendix B

**Table A2:** Results of all patients’ questionnaires (n = 48).

Questions	Fully Agree		Rather Agree		Partly/ Partly		Rather Not Agree		Strongly Disagree	
	n	%	n	%	n	%	n	%	n	%
The video consultation helped me to ease my problem.	8	16.7	14	29.2	14	29.2	4	8.3	1	2.1
I feel well advised.	26	54.2	10	20.8	10	20.8	1	2.1	0	0.0
I felt well understood regarding my concerns.	27	56.3	13	27.1	5	10.4	0	0.0	1	2.1
The use of video consultation technology is a useful addition to face-to-face counselling.	28	58.3	14	29.2	4	8.3	1	2.1	0	0.0
The video consultation can replace the personal consultation with the dermatologist.	9	18.8	11	22.9	22	45.8	5	10.4	0	0.0
I felt uncomfortable during the video consultation.	0	0.0	4	8.3	5	10.4	12	25.0	22	45.8
I would like to continue using digital services, e.g., video consultations/online seminars.	22	45.8	12	25.0	9	18.8	1	2.1	2	4.2
During the video consultation, the dermatologist was able to get a good picture of my skin condition.	15	31.3	10	20.8	11	22.9	8	16.7	2	4.2
I was able to explain my (medical) problems well enough during a video consultation.	28	58.3	14	29.2	3	6.3	1	2.1	0	0.0
The lack of physical examination during video consultation is a problem.	3	6.3	5	10.4	21	43.8	10	20.8	6	12.5
It bothered me that the dermatologist could not touch/feel my skin/skin lesions during the video consultation.	1	2.1	4	8.3	13	27.1	19	39.6	9	18.8
My privacy was protected during the video consultation.	34	70.8	7	14.6	0	0.0	0	0.0	0	0.0
The conversation with the dermatologist during the video consultation is just as satisfying as talking to him/her in person.	18	37.5	18	37.5	10	20.8	0	0.0	1	2.1
Video consultations make it easier for me to contact the dermatologist.	21	43.8	12	25.0	11	22.9	0	0.0	0	0.0
Video consultations are a convenient form of health care for me.	17	35.4	13	27.1	13	27.1	3	6.3	0	0.0
Video consultations save me time.	32	66.7	14	29.2	0	0.0	0	0.0	0	0.0
I was satisfied with the video consultation I had with the dermatologist.	33	68.8	9	18.8	4	8.3	0	0.0	0	0.0
I would feel much more comfortable with a face-to-face conversation.	3	6.3	5	10.4	25	52.1	6	12.5	7	14.6
The video consultation technique worked.	30	62.5	6	12.5	7	14.6	4	8.3	1	2.1
The video consultation technology is easy to use.	26	54.2	12	25.0	5	10.4	3	6.3	0	0.0
My technical equipment made it difficult to understand the dermatologist.	1	2.1	2	4.2	4	8.3	10	20.8	30	62.5
The devices made me feel uncomfortable.	0	0.0	1	2.1	4	8.3	11	22.9	28	58.3
I could see the dermatologist well.	33	68.8	10	20.8	1	2.1	0	0.0	2	4.2
I could hear the dermatologist well.	35	72.9	9	18.8	0	0.0	1	2.1	1	2.1
I have an internet connection which, in principle, allows me to participate in video consultations.	40	83.3	5	10.4	2	4.2	0	0.0	0	0.0
The internet connection was stable during the video consultation.	38	79.2	6	12.5	2	4.2	0	0.0	1	2.1
The information sheet helped me to get to terms with the video consultation technique.	26	54.2	9	18.8	8	16.7	1	2.1	0	0.0

## Appendix C

**Table A3:** Results of all dermatologists’ questionnaires (n = 62).

Questions	Fully Agree		Rather Agree		Partly/ Partly		Rather Not Agree		Strongly Disagree	
	n	%	n	%	n	%	n	%	n	%
The video consultation helped the patient to ease his/her problem.	21	33.9	24	38.7	14	22.6	2	3.2	1	1.6
I was able to advise the patient in a targeted manner.	21	33.9	29	46.8	9	14.5	3	4.8	0	0.0
I was able to provide comprehensive advice to the patient.	22	35.5	21	33.9	11	17.7	5	8.1	3	4.8
I was able to fulfil my medical duty of care.	18	29.0	15	24.2	15	24.2	11	17.7	2	3.2
The use of video consultation technology is a useful addition to face-to-face counselling.	23	37.1	18	29.0	9	14.5	10	16.1	1	1.6
The video consultation can replace the personal consultation with the patient.	14	22.6	9	14.5	10	16.1	19	30.6	10	16.1
I was not comfortable doing the counselling via a video conferencing system (BigBlueButton).	2	3.2	3	4.8	19	30.6	17	27.4	18	29.0
I would like to continue offering digital services, e.g., video consultations/online seminars.	15	24.2	15	24.2	18	29.0	13	21.0	1	1.6
The lack of physical examination during video consultation is a problem.	30	48.4	18	29.0	12	19.4	1	1.6	1	1.6
It bothered me that I could not touch/feel the skin/skin lesions of the patient during the video consultation.	29	46.8	18	29.0	8	12.9	3	4.8	4	6.5
The privacy of the patient is protected during the video consultation.	21	33.9	28	45.2	10	16.1	0	0.0	0	0.0
The conversation with the patient during the video consultation is just as effective as a personal conversation.	19	30.6	10	16.1	19	30.6	9	14.5	5	8.1
Video consultations make it easier for me to contact the patient.	22	35.5	23	37.1	15	24.2	2	3.2	0	0.0
Video consultations are a convenient form of health care for me.	20	32.3	19	30.6	13	21.0	9	14.5	1	1.6
Video consultations save me time.	22	35.5	4	6.5	16	25.8	12	19.4	8	12.9
I was satisfied with the video consultation I had with the patient.	7	11.3	24	38.7	20	32.3	6	9.7	4	6.5
I would feel more comfortable with a face-to-face conversation.	15	24.2	21	33.9	14	22.6	6	9.7	6	9.7
The video consultation technique worked.	25	40.3	17	27.4	15	24.2	2	3.2	2	3.2
The video consultation technology is easy to use.	33	53.2	22	35.5	6	9.7	0	0.0	0	0.0
My technical equipment made it difficult to understand the patient.	2	3.2	1	1.6	1	1.6	20	32.3	36	58.1
Due to the technical equipment of the patient, I had difficulties hearing him/her.	5	8.1	6	9.7	2	3.2	15	24.2	32	51.6
The devices made me feel uncomfortable.	1	1.6	2	3.2	8	12.9	12	19.4	34	54.8
I could see the patient well.	22	35.5	10	16.1	5	8.1	17	27.4	6	9.7
I could clearly see the skin condition of the patient.	2	3.2	6	9.7	5	8.1	11	17.7	37	59.7
The internet connection was stable during the video consultation.	33	53.2	18	29.0	1	1.6	3	4.8	5	8.1
I could hear the patient well.	32	51.6	18	29.0	3	4.8	5	8.1	2	3.2
In general, I am satisfied with the video consultation system.	3	4.8	15	24.2	25	40.3	12	19.4	6	9.7

## Appendix D

**Table A4:** Translated results of all open-ended questions.

Patients	Dermatologists
**What will you tell others about the counselling?**	
A good alternative if you have to travel a long way.	
A great alternative to visiting the doctor in person, especially if you live far away. I can recommend it without hesitation.	
I had a good conversation with my dermatologist, but it’s a lot more work (travel, parking).	
Yes.	
The video consultation is to be recommended.	
Much better than driving long distances. No waiting times. Super.	
In general very pleasant, no long journey. Comfortable from home.	
The consultation was very good and the dermatologist had an informative conversation with me, I felt very well understood and advised. Unfortunately, the picture was a bit “crispy” which made it difficult for the dermatologist to make an accurate diagnosis and he was therefore partly dependent on my verbal information. In my opinion, a palpation report is also very important in order to check the exact condition of the skin. The time saved and the fact that I didn’t have to go all the way to the University Hospital in Osnabrück was very pleasant. All in all, this type of virtual visit is a useful addition, but only an addition to improve the density of contact between doctor and patient. This type of visit cannot completely replace personal contact between dermatologist and patient, especially in the case of skin diseases, physical findings (e.g., palpation) are, in my opinion, very important.	
Great thing saves time and money.	
The consultation was simple and uncomplicated, I had sent my dermatologist photos 2 days beforehand, which made the consultation even easier.	
Everything went flawlessly. Worked without any problems.	
The online physician follow-up consultation via webcam made it possible for me to be in “personal contact” with my treating physicians of the iDerm team, even though I am over 700 km away. I find it very good that this is possible with technology, as my current questions could be answered directly.	
That the treatment has been successful for me and I have learned a lot.	
I can only report good things.	
Only good.	
It was good and served its purpose.	
Everything worked out fine.	
The consultation is almost as if the doctor is sitting in front of you.	
Everything went well, except for the tone at the beginning.	
I like the fact that there was an online check-up of my skin condition. My questions that came up while I was working again could be answered by the physicians on the phone/laptop. A consultation via laptop is a good thing if the circumstances allow it, but of course I find a physician’s presentation better.	
That the video consultation was top notch.	
Actually, nothing concrete…I wouldn’t tell you any more if I had sat with the doctor in person.	
Everything went well.	
Was good.	
Everything was very good.	
It is a good alternative for follow-up treatment, but not for the main examinations.	
The consultation was good, but I couldn’t handle the technology.	
It’s a good thing and saves time than having to go there every time.	
It was very interesting and informative.	
The advice was good, but I couldn’t handle the technology.	
**What do you think could be done better?**	
Possibility of a technical trial before the physician arrives.	The quality of the image should be higher.
Everything went well for me, no suggestions for improvement.	Unfortunately, it is not possible to assess the skin findings well. The scaling described was only partially visible.
I don’t know anything now.	The image quality was insufficient. No assessment of the skin condition was possible.
My light incidence was bad for skin assessment (skylight without blackout,) needs to be darker and a lamp to go with it.	Better image quality.
Nothing.	Better image quality for the assessment of the skin condition.
One should be made more aware of the video consultation option—unfortunately not offered until asked.	The image quality could be better.
I would have liked to do the video conference with the tablet because of the size. Unfortunately, the programme wanted a platform that was not supported by web browsers, Safari or Microsoft, although these are the most common platforms?	The quality of the image should be better in order to be able to better assess the skin condition, one is dependent on the assistance of the insured person as to where which skin changes are.
I would rate the procedure as very good, with the restriction that the physical findings were missing and the image was “crispy” (this was certainly due to my internet access). The physician exploited the potential of this virtual visit very well, but as already mentioned above, it can completely replace a face-to-face visit in my opinion.	It was good that the patient had sent her own photos in advance by email. The patient did not have a headset. As a result, I had great difficulty understanding the echo phenomenon and because simultaneous conversation was not possible.
Everything went very well.	The image quality should be better.
it went well, so there is nothing to improve.	Due to the fact that the skin findings had almost healed, the quality of the transmission of the skin findings was not so decisive. The insured person showed residual scaling that I would not have seen without his help. The quality of the assessment of the skin findings could be better.
Picture quality, but the transmission rate is probably less reliable.	Quality of the images for the assessment of the skin findings.
I have no suggestions for improvement, everything was well organized.	The patient was only marked with a number and not his name in the waiting area. I took over the patient and did not see him myself beforehand. I had to ask first whether this was really the right patient. The picture quality was very poor, and the picture and sound were often not very clear.
Leisure activities could be more, the food could be better and healthier, I put on a lot of weight.	Better image quality, you have to rely on the patient’s cooperation, the patient has to show you where inflammatory skin changes are.
Actually nothing, it was really good the way it was.	The image quality is insufficient, without the patient’s help I would not know where they are.
Everything was OK.	After the video consultation, the insured person sent photos on which the skin condition could be seen and assessed much better than in front of the camera. Photos should be an integral part of the video consultation.
The technology worked, but the physician couldn’t understand me very well, a matter of attitude? A few weeks ago, with the other physician, the same conversation went perfectly with the same technology.	Improved image quality to better assess skin condition. Patients have to show you where skin lesions are and then you can at best guess them.
Nothing.	Image quality.
There was no sound at the beginning. But the physician fixed the error.	Patient could not be heard well—therefore telephone contact with camera. Assessment of skin condition passably possible, but less precise than in clinical examination with direct patient contact.
No suggestion. I found the reminder of the appointment one day before sufficient.	Skin findings were hardly to be seen. Mostly overexposed. Sound interruptions meant that I had to ask several times.
Nothing at all it was all to my complete satisfaction.	Better image quality.
It was my first video consultation…after we had solved the first small technical problems, everything ran smoothly and satisfactorily.	The quality of the image, the skin findings could only be ascertained with the help of the patient.
It was good, but I don’t see any improvement.	I could only understand individual words from time to time, so I called the insured and communication took place over the phone. The skin findings could hardly be made this time due to the poor image quality, I was dependent on the patient’s help (much worse than in other video consultations).
Everything was fine.	Image quality.
Nothing at all, everything was fine.	Skin condition was not visible.
	Quality of the internet connection. The e-mail link opens the normal Internet Explorer, here a black screen appears. You have to choose another explorer yourself for a functioning consultation.
	Video consultation cannot fully replace the dermatological examination.
	The quality of the image transmission
	Better image quality
	Better image quality. Better support for patients regarding the technique in advance.
	Better image quality. Better technical support for patients in advance.
	Better image quality.
	The insured could not understand me, so communication was by phone, images were sufficiently good.
	Better image quality. Technology easier to use for the patient.
	Better image quality. Easier to use for the insured person.
	The assessment is based on the fact that I had photos of the insured person.
	During the conversation I could hear myself twice, this was very unpleasant and could not be changed by dialling in again. Attempting to make a phone call was also not possible, as the insured person was using her mobile phone for the video conference and a conversation via telephone was not possible. Picture quality was mediocre.
	Poor picture quality, the insured person sent photos by e-mail for a better assessment.
**What did you particularly like?**	
Easy to use BigBlueButton.	Communication and eye contact worked well.
Uncomplicated, not time-consuming, I am thrilled.	Communication worked well.
That I didn’t have to drive 130 km there and back.	Technology and communication worked well, quick contact with the insured person.
Easy to use.	Technical support from the Teledermos team.
Not having to drive to Osnabrück.	Everything else, especially the technology and communication, worked well.
Easy to use.	Fast and yet personal communication.
No waiting times. No travelling.	Communication.
Consultation from home.	Time saving, technology, communication.
The physician’s advice and his successful effort to compensate for the disadvantages of a video consultation, but above all his trust in my information.	Support by the TeleDermos team in case of technical problems.
The good picture and sound quality.	Time saving.
That it was so easy.	Communication.
Time saving/money saving (petrol)/well-functioning technology. The physician Mrs. X [Name of the physician anonymized by the authors.], very friendly and good looking.	Overall uncomplicated system.
That the technology worked so well.	Uncomplicated contact. Important information could be requested.
All the staff and physicians were very nice.	Direct communication.
That I can see the physician at the video consultation.	Good communication.
Conveniently from home.	Unfortunately, nothing this time.
That you can still describe and, if necessary, also show problems in the short official channels, so to speak in private.	Communication, short distances, time saving.
Relaxed consultation from home.	Advice well possible. Would also have been possible by phone, but more personal through the camera.
That the communication worked so well.	Unproblematic operation, quick help with start-up difficulties :-)
The physician was very nice and friendly.	Communication, time saving.
That the physicians also took a lot of time online for the follow-up consultation (20 min).	Short direct communication.
Super picture quality and super sound quality.	To give patients the opportunity to be followed up by me, even if they come from far away.
It was like a “personal” physician consultation…but with a distance of 120 km.	
Time saved.	
Nothing.	
It was pretty quick.	
The medical consultation was good, even without picture and sound.	
That everything worked out well with the video consultation.	
There is no waiting time the appointment is fixed.	
The medical consultation was good, even without picture and sound.	
**What else would you like to tell us?**	
I am very satisfied with my dermatologist.	
No.	
In order to increase the density of physician-patient contact and to ensure optimal monitoring of the course of the disease, the online visit is certainly very good, especially for patients with physical limitations I see good potential. However, regular presence visits should take place. That is why I have decided for the next visit in Osnabrück.	The first appointment with the patient could not take place due to the patient’s lack of a suitable browser. At the end of the following 2nd video consultation with the suitable browser, the patient could no longer hear me (unclear why), so we then spoke on the phone.
No.	The skin findings are difficult to assess, the patient’s assistance is necessary.
---	Many thanks for the support :-).
I will keep the three inpatient weeks in good memory.	At the beginning, the connection could not be established. It was necessary to dial in again twice, but then it worked.
I felt well looked after in the iDerm during my rehab. My skin problems were taken very seriously every day, everything was treated and therapized very well. Thank you. I found the seminars very informative and exciting, there could be a few more.	The browser on the patient’s PC was not suitable, so the video consultation took place via the smartphone. Language communication was good, especially for the patient less effort (travelling). However, the quality of the recording was so poor that the skin findings could not be ascertained.
☺No, I am completely satisfied.	Due to technical difficulties on the part of the patient, a telephone call was made instead of the video consultation.
No.	Additional photos sent by e-mail are very helpful.
No.	Some of the skin findings are much easier to recognize on the photos sent.
I think it is a good aftercare, also how to continue professionally.	The skin findings could be better assessed on the photos sent in parallel by the patient by e-mail.
No.	In this case, the contact was satisfactory because only the hands were affected, which the patient had photographed well, and because there was no momentum and the findings had improved since discharge. In other patients with a more complicated course of the disease and several affected localizations, my judgement would have been more critical.
I am very satisfied, I would like to do it again…but sometimes a personal visit is unavoidable.	
Everything is great at DermOS.	
No.	
No.	
